# Long-term efficacy, safety, and tolerability of a subcutaneous immunoglobulin 16.5% (cutaquig^®^) in the treatment of patients with primary immunodeficiencies

**DOI:** 10.1093/cei/uxac092

**Published:** 2022-10-08

**Authors:** Roger H Kobayashi, Jiří Litzman, Isaac Melamed, J Fernando Mandujano, Ai Lan Kobayashi, Bruce Ritchie, Bob Geng, T Prescott Atkinson, Syed Rehman, Sonja Höller, Eva Turpel-Kantor, Huub Kreuwel, J C Speer, Sudhir Gupta

**Affiliations:** UCLA School of Medicine, Los Angeles, CA, USA; Department of Clinical Immunology and Allergology, St Anne’s University Hospital in Brno, Faculty of Medicine, Masaryk University, Brno, Czechia; IMMUNOe Research Center, Centennial, CO, USA; Pediatric Pulmonary Associates of North Texas, Frisco, TX, USA; Midlands Pediatrics, Papillion, NE, USA; Division of Hematology, Department of Medicine, University of Alberta Hospital, Edmonton, AB, Canada; Divisions of Allergy and Immunology, University of California San Diego, La Jolla, CA, USA; Department of Pediatric Allergy, Asthma and Immunology, University of Alabama, Birmingham, AL, USA; Allergy and Asthma Center Inc., Toledo, OH, USA; Octapharma Pharmazeutika Produktionsges.m.b.H., Vienna, Austria; Octapharma Pharmazeutika Produktionsges.m.b.H., Vienna, Austria; Octapharma US, Paramus, NJ, USA; Octapharma US, Paramus, NJ, USA; Division of Basic and Clinical Immunology, University of California Irvine, Irvine, CA, USA

**Keywords:** long-term safety, prospective data, primary immunodeficiencies, subcutaneous immunoglobulin, tolerability

## Abstract

A prospective study and its long-term extension examined whether weekly treatment of patients with primary immunodeficiencies (PIDs) with a 16.5% subcutaneous immunoglobulin (SCIg; cutaquig^®^) confers acceptable efficacy, safety, and tolerability over a follow-up of up to 238 weeks (>4 years). Seventy-five patients received 4462 infusions during up to 70 weeks of follow-up in the main study and 27 patients received 2777 infusions during up to 168 weeks of follow-up in the extension. In the main study, there were no serious bacterial infections (SBIs), and the annual rate of other infections was 3.3 (95% CI 2.4, 4.5). One SBI was recorded in the extension, for an SBI rate of 0.02 (upper 99% CI 0.19). The annual rate of all infections over the duration of the extension study was 2.2 (95% CI 1.2, 3.9). Only 15.0% (1085) of 7239 infusions were associated with infusion site reactions (ISRs), leaving 85.0% (6153) of infusions without reactions. The majority of ISRs were mild and transient. ISR incidence decreased over time, from 36.9% to 16% during the main study and from 9% to 2.3% during the extension. The incidence of related systemic adverse events was 14.7% in the main study and 7.4% in the extension. In conclusion, this prospective, long-term study with cutaquig showed maintained efficacy and low rates of local and systemic adverse reactions in PID patients over up to 238 weeks of follow-up.

## Teaser

Over the whole main study period, 77.7% of the 4,462 infusions resulted in no infusion site reactions (ISRs). In the extension study, most of the 2,777 infusions (96.5%) induced no ISRs. In both adult and pediatric patients, mild ISRs were by far the most common, and in children mild ISRs made up 96% of local reactions.

## Introduction

Primary immunodeficiencies (PIDs) are a diverse group of genetic aberrations of the immune system that result in an increased risk of recurrent bacterial and viral infections. These can often lead to more serious complications such as organ failure and even death if not managed appropriately [1–5]. For patients with primary antibody deficiencies, long-term immunoglobulin (Ig) replacement therapy, delivered either intravenously (IV), or subcutaneously (SC), may be required to prevent infections and their complications [2].

SCIg is administered at more frequent intervals and at higher cumulative doses than IVIg to achieve comparable IgG plasma levels that remain stable between infusions. SCIg is also associated with a reduced risk of systemic adverse reactions than IVIg and can be self-administered or administered by a caregiver at home, which may be preferred by some patients and physicians over IVIg [6–8].

Cutaquig^®^ (Octapharma AG), a 16.5% solution of human Ig for SC administration, is approved for use in the USA, Canada, Europe, and Australia. The lower viscosity of a 16.5% product compared with a 20% product results in improved injectability in the form of less force required and easier handling during administration [9]. Cutaquig is based on the manufacturing process of Octagam® and is stabilized with maltose.

People with more severe PIDs may require long-term SCIg treatment, but in the clinic, extensive formalized, long-term monitoring is often not feasible due to patients’ non-adherence to follow-up schedules (e.g. forgetfulness, transportation difficulties, and other obligations) and financial or insurance issues. Therefore, it is important to examine whether the efficacy and safety profile of SCIg remains acceptable and whether repeated self-administration remains practical and tolerated over extended periods of time. The majority of reported pivotal studies of SCIg have included approximately one-year follow-up [10–12] and published long-term data are sparse.

The current analysis presents final data from a pivotal Phase 3 study with cutaquig in patients with PID (NCT01888484) and its long-term extension (NCT03907241). The aim of this analysis is to compare the data from the extension with the primary study to determine whether there are differences in the safety parameters and clinical outcomes over long-term treatment with cutaquig. Together, the two studies investigated the efficacy, safety, and tolerability of cutaquig in adult and pediatric patients with PID, with a unique long-term follow-up of more than four years in ~50% of the patients.

## Methods and materials

### Study design

**Figure 1:**
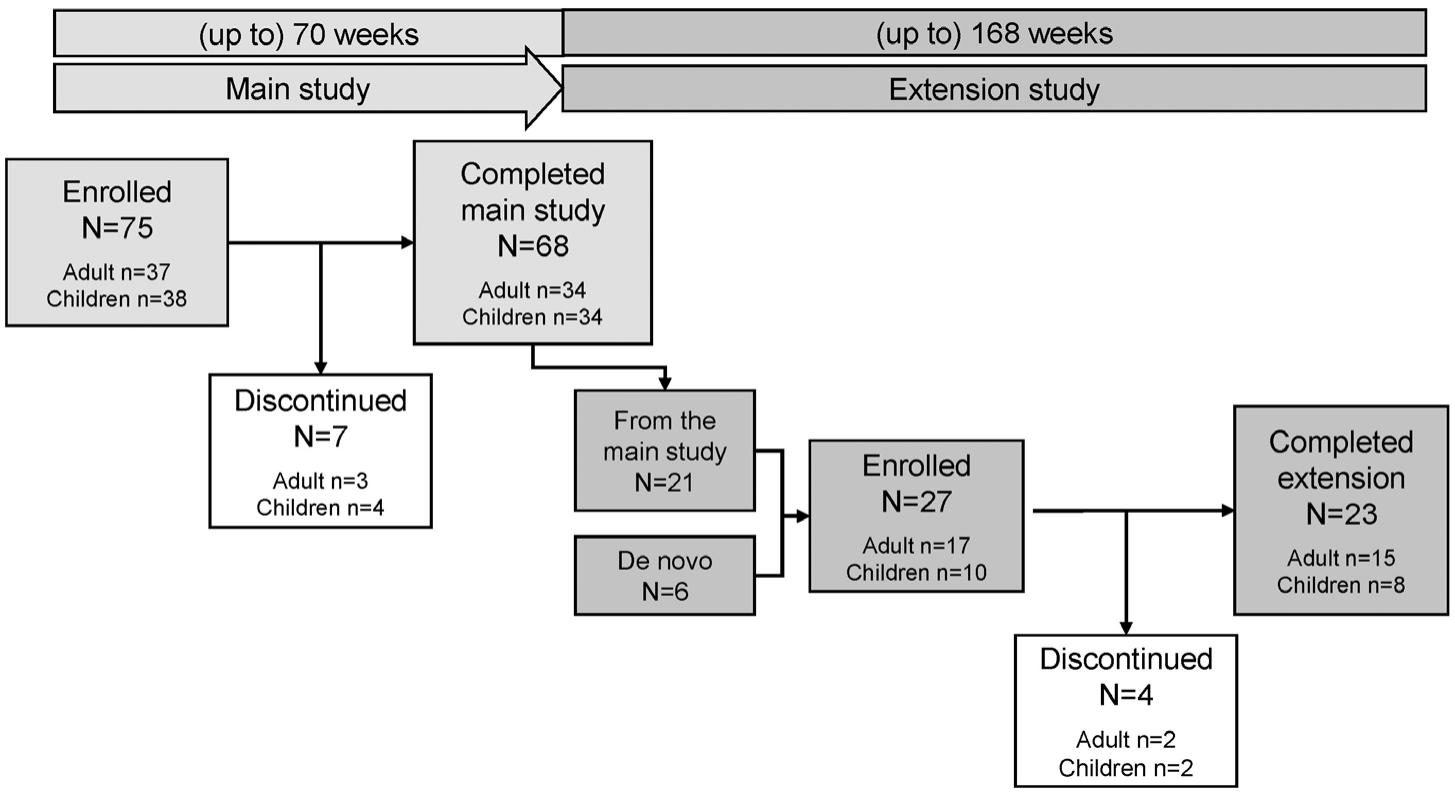
Study design and patient disposition.

The results reported here are from the final analysis of the pivotal study with cutaquig (SCGAM-01) and its long-term extension (SCGAM-03) (Fig. 1). The main study was conducted at 18 centers in Europe, USA, and Canada, and the extension study at seven centers in USA and Canada. In the USA, only patients who completed the main study continued infusions in the extension study, while at the Canadian site, only de novo patients who were on SCIg treatment for at least 12 months prior were enrolled in the extension.

The details of the main study design have been published previously [13]. Both studies were prospective, open-label, non-controlled, single-arm, multicenter Phase 3 studies. The main study consisted of a 12-week wash-in/wash-out period followed by a 12-month efficacy evaluation period. To collect long-term data, the patients who completed the main study had the opportunity to enter the long-term extension. Patients who completed the main study before the start of the extension study were allowed to continue IgG treatment with any commercially available SCIg preparations until the time when they could be treated within the extension study. Follow-up was up to 70 weeks in the main study and up to 168 weeks in the extension, for a total of up to 238 weeks (Fig. 1).

The main inclusion criteria were a diagnosis with a PID [as defined by the European Society for Immunodeficiencies (ESID) and Pan-American Group for Immunodeficiency (PAGID)] or primary IgG deficiency (defined as depressed antibody levels below 2 standard deviations of the mean for age-matched standard controls) with clinically significant immunodeficiency [14, 15]. To enter the extension study, patients in the US sites were to have completed the main study with good tolerance of cutaquig (as determined by the investigator) and to be willing to continue long-term treatment and follow-up. At the Canadian site, patients were eligible to enter the extension if they were between 18 and 75 years old, with PIDs (as defined by ESID and PAGID) and if they required Ig replacement therapy due to hypogammaglobulinemia or agammaglobulinemia and were currently receiving SCIg treatment.

All patients enrolled in the main study had been previously receiving regular IVIg treatment with constant dosing for at least six prior infusions. In the study, they received weekly cutaquig doses 1.5 times their previous monthly IVIg dose split into four equal weekly SCIg infusions, as described previously [13]. In the extension, patients resumed the same dose of cutaquig they received at the week 64 infusion of the main study. Newly enrolled patients received the same dose (in mg/kg/week) as their previous commercial SCIg product.

Patients/caregivers were trained to administer cutaquig and were instructed on measures to be taken in case of severe adverse events (AEs). In addition, they were provided a patient diary in which they documented parameters related to the infusion (date, lot numbers, and infusion rate), efficacy (occurrence of infections, missed days from work/school/daycare, inpatient hospital stays), tolerability (local tissue reactions), and health-related quality of life (QoL) [assessed using Child Health Questionnaire-Parent Form, 50 items (CHQ-PF50) for patients <14 years old and the Short Form Health Survey, 36 item (SF-36) for patients ≥14 years old].

A QoL questionnaire was administered at the first visit and again at the patient’s last visit, which occurred 1 week after their last infusion. For the QoL assessments in the extension, patients were administered the same questionnaire as in the main study. That is, parents or guardians of patients who were below 14 years of age when they entered the main study continued using the CHQ-PF50. Investigators graded the severity (mild, moderate, or severe) and seriousness (non-serious or serious) of AEs and assessed causality. At each clinic visit, samples were collected for laboratory assessments before the administration of cutaquig. IgG trough levels were analyzed at local laboratories according to the site’s standard procedures.

Efficacy and safety results were analyzed by age groups: younger children (≥2 and <6 years), older children (≥6 and <12 years), adolescents (≥12 and <17 years), and adults (≥17 years). Patients enrolled in the extension study were analyzed in the same age group that they were in originally, irrespective of whether their age group changed.

A pharmacokinetic (PK) study was conducted in a subset of patients in the main study (*n* = 37; 18 children and 19 adults) to determine the dose conversion factor (DCF) from IVIg to SCIg. Patients participating in the PK substudy received a single dose of their previous IVIg at enrollment into the main study. To establish the PK profile, IgG trough levels were measured at three time points: after the last IVIg administration (PK_IV_), at the end of the 12 week wash-in/washout phase, after the 11th infusion (PK_SC1_), and after the 28th SCIg administration (PK_SC2_). The DCF was calculated by taking the area under the curve (AUC) of IVIg before switching to cutaquig and dividing it by the AUC of SCIg after the 28th infusion of cutaquig. Bioequivalence was assessed by taking the ratio of the AUC standardized to 1 week (AUCτ) for PK_SC2_ and PK_IV_.

A simulation-based analysis (population PK [popPK] analysis) was performed to assess the impact of different SCIg dosing regimens on plasma Ig concentrations. Specifically, it was used to simulate IgG trough concentrations at steady state in order to assess the risk for trough concentrations falling below 5 g/l for DCFs of 1.0 and 1.4 and various dosing regimens ranging from daily to every 4 weeks.

An Independent Data Monitoring Committee (IDMC) reviewed laboratory and clinical data sets with particular attention to thromboembolic events and clinically significant hemolysis at various intervals during the study. Both studies were carried out in accordance with the recommendations of the US Food and Drug Administration (FDA) Guidance for Industry for IgG Studies and European Medicines Agency (EMA) guideline on the clinical investigation of SCIg and the international standards of Good Clinical Practice. All patients gave written informed consent in accordance with the Declaration of Helsinki. The protocols were approved by the National Competent Authorities, International Ethics Committees and Institutional Review Boards (IRBs, with two central IRBs used in the US).

### Statistical analysis

All data collected were summarized and presented descriptively. Missing data were not imputed except for missing body weight, where the last available weight measurement was used for calculating the dose per kilogram of body weight. Whenever appropriate, analyses were stratified according to the predefined age groups from the main study. For these studies, serious bacterial infections (SBIs) were defined as bacterial pneumonia, bacteremia/sepsis, osteomyelitis/septic arthritis, visceral abscess, or bacterial meningitis. The rate of SBI per person-year during the treatment period with cutaquig was presented as point estimates of the rate along with a one-sided 99% confidence limit.

The primary efficacy objective of the main study was to evaluate the efficacy of cutaquig in preventing SBIs. The primary objective of the extension was to assess the long-term safety and tolerability of cutaquig. To this end, the following were recorded: incidence of treatment-emergent adverse events (TEAEs), local ISRs, vital signs (blood pressure, pulse, body temperature, and respiratory rate), and laboratory parameters (hematology, clinical chemistry, basic urinalysis, and tests for viral safety). The occurrence of TEAEs was characterized and analyzed by seriousness, severity, and relationship to cutaquig. ISRs and infection-related AEs were distinguished from other AEs and were characterized and analyzed separately. ISRs were analyzed by the number of infusions with ISRs by severity (on a scale from 0 [none] to 4 [severe]), type of reaction, and study week. Efficacy was evaluated by the occurrence of SBIs and the annual rate of all infections, and QoL by the CHQ-PF50 questionnaire in participants under the age of 14 years and SF-36 for those 14 years and older.

## Results

### Patient demographics and baseline characteristics

The main study enrolled 75 patients and the extension enrolled 27 patients (21 patients from the main study and 6 de novo). The breakdown of patients by age in the main study was as follows: 12 younger children (≥2 and <6 years), 14 older children (≥6 and <12 years), 12 adolescents (≥12 and <17 years), and 37 adults (≥17 years). The patients’ ages ranged from 2 to 73 years and the overall mean (SD) age was 27.8 (21.29) years. The median age was 16 years. The extension enrolled a markedly older population, including 10 children (2 younger children, 4 older children, and 4 adolescents) and 17 adults. The mean (SD) age for the extension was 39.3 (24.4) years, with a median of 51 years. The etiology of PID for most patients was common variable immunodeficiency (CVID) and X-linked agammaglobulinemia (XLA) (Table 1).

**Table 1:** Baseline characteristics

	Children (≥2 to <17 years)		Adults (≥17 years)		Total	
	Main study (*n* = 38)	Extension (*n* = 10)	Main study (*n* = 37)	Extension (*n* = 17)	Main study (*N* = 75)	Extension (*N* = 27)
Sex [*N* (%)]						
Male	29 (76.3)	6 (60.0)	10 (27.0)	4 (23.5)	39 (52.0)	10 (37.0)
Female	9 (23.7)	4 (40.0)	27 (73.0)	13 (76.5)	38 (48.0)	17 (63.0)
Age [mean ± SD (range)], years	8.7 ± 4.2 (2–16)	9.4 ± 3.7 (5–14)	47.5 ± 13.6 (20.0–73.0)	56.1 ± 11.9 (25.0–73.0)	27.8 ± 21.9 (2–73)	39.3 ± 24.4 (6–73)
Body weight [mean ± SD (range)], kg	36.4 ± 18.9 (13.0–86.4)	50.5 ± 28.3 (22.7–97.0)	68.7 ± 12.7 (44.3–98.6)	72.1 ± 14.2 (47.7–101.0)	52.37 ± 22.8 (13.0–98.6)	64.07 ± 22.7 (22.7–101.0)
Etiology of PID [*N* (%)]						
CVID	20(52.6)	10 (100)	36 (97.3)	14 (82.4)	56 (74.7)	24 (88.9)
XLA	6 (15.8)	0	0	0	6 (8.0)	0
Other^*^	12 (31.6)	0	1 (2.7)	3 (17.6)	13 (17.3)	3 (11.1)
Region [*N* (%)]						
Europe	23 (60.5)	0	17 (45.9)	0	40 (53.3)	0
United States	15 (39.5)	10 (100)	17 (45.9)	11 (64.7)	32 (42.7)	21 (77.8)
Canada	0	0	3 (8.1)	6 (35.3)	3 (4.0)	6 (22.2)

*Other: two cases of hypogammaglobulinemia, and one case each of agammaglobulinemia (not X–linked), selective deficiency of IgG1 and IgG2 with deficiency of specific antibodies; two cases of DiGeorge syndrome, and one case each of hyper IgM hyper IgM syndrome, X-linked hyper IgM syndrome, Nijmegen breakage syndrome, hypogammaglobulinemia IgG1, and Wiskott–Aldrich syndrome.

Abbreviations: CVID, common variable immunodeficiency; XLA, X-linked agammaglobulinemia.

The mean duration of follow-up in the main study was 60 weeks (up to 70 weeks) and in the long-term extension, it was 104 weeks (up to 168 weeks). The distribution of the duration of follow-up for patients who entered the extension study was as follows: 12/27 (44.4%) longer than 4 years (>208 weeks) over both studies; 4/27 (14.8%) between 3 and 4 years over both studies; 5/27 (18.5%) between 1.5 and 3 years over both studies and; 6/27 (22.2%) less than one year (de novo patients). The six de novo patients were followed up for an overall shorter length of time, but their inclusion does not markedly affect the overall length of follow-up.

Seven patients (four adolescents and three adults) withdrew from the main study and 4 patients (two children and two adults) from the extension. The most common reasons for withdrawals were the length of infusion (three patients) and a preference for IVIg (two patients). No withdrawal was due to AEs or ISRs.

### SCIg administration characteristics

Seventy-five patients received a total of 4462 infusions in the main study and 27 patients received 2777 infusions throughout the extension study, for a total of 7239 infusions. The mean dose of cutaquig administered was 0.16 g/kg/infusion in children (≥2 and <17 years) and 0.19 g/kg/infusion in adults (≥17 years) in the main study, and 0.17 g/kg/infusion in both children and adults in the extension. While the dosing did not change markedly from the main study to the extension, in adults the maximum infusion flow rate increased from a mean (SD) of 60.4 (17.5) ml/h to 91.9 (28.3) ml/h and consequently the mean duration of infusion decreased from 113.3 (48.4) min to 76.3 (39.8) min (Table 2). The maximum infusion flow rate per patient per site was 52 ml/h/site in adults and 25 ml/h/site in children.

**Table 2:** Dosing characteristics, infusion volumes, and infusion flow rates

	Children (≥2 to <17 years)		Adults (≥17 years)		Total	
	Main study (n = 38)	Extension (*n* = 10)	Main study (*n* = 37)	Extension (*n* = 17)	Main study (*N* = 75)	Extension (*N* = 27)
Overall number of infusions (*n*)	2213	1070	2249	1707	4462	2777
Dose administered (g/kg BW/patient)	0.16 ± 0.05 (0.06–0.29)	0.17 ± 0.08 (0.06–0.30)	0.19 ± 0.07 (0.09–0.39)	0.17 ± 0.07 (0.07–0.38)	0.17 ± 0.07 (0.06–0.39)	0.17 ± 0.08 (0.06–0.38)
Average duration of infusion (active time only) [min]	84.2 ± 45.9 (29.9–230.5)	82.6 ± 28.4 (37.4–126.5)	113.3 ± 48.4 (55.6–304.5)	76.4 ± 39.8 (11.5–130.1)	98.5 ± 49.1 (29.9–304.5)	78.7 ± 35.6 (11.5–130.1)
Infusion volumes and flow rates per patient						
Maximum volume administered (ml) weekly	38.4 ± 20.2 (8.0–90.0)	57.9 ± 29.5 (13.0–102.0)	78.9 ± 31.6 (36.0–159.0)	75.3 ± 38.1 (24.0–150.0)	58.4 ± 33.2 (8.0–159.0)	68.9 ± 35.6 (13.0–150.0)
Maximum infusion flow rate (ml/h)	39.4 ± 11.1 (15.0–51.8)	42.2 ± 12.9 (20.0–60.0)	60.4 ± 17.5 (29.4–97.9)	91.9 ± 28.3 (45.0–130.9)	49.8 ± 17.9 (15.0–40.0)	73.5 ± 33.9 (20.0–130.9)
Maximum infusion flow rate/site (ml/h/site)	18.7 ± 6.0 (3.8–30.0)	17.2 ± 4.9 (9.4–25.0)	22.8 ± 9.5 (10.0–51.8)	27.0 ± 8.3 (11.3–43.6)	20.7 ± 8.1 (3.8–51.8)	23.4 ± 8.6 (9.4–43.6)

Data are mean ± SD, (range) except where indicated.

Abbreviations: BW, body weight.

### Efficacy

There were no incidences of SBI in the main study. In the extension study, a 73-year-old patient experienced bacteremia, which was recorded as an SBI but was not related to the study drug. The patient experienced fever, chills, and exhaustion, and upon examination her blood cultures revealed *Escherichia coli* bacteremia, which was recorded as a severe and serious event. She was hospitalized for 3 days and treated with antibiotics. The event resolved within 5 days and her treatment with cutaquig was not interrupted. The rate of SBI per person-year in the extension study was therefore 0.02, with the upper 99% CI of 0.19 (Table 3).

**Table 3:** Summary of efficacy results

	Children (≥2 to <17 years)		Adults (≥17 years)		Total	
	Main study (*n* = 38)	Extension (*n* = 10)	Main study (*n* = 37)	Extension (*n* = 17)	Main study (*N* = 75)	Extension (*N* = 27)
Total number of patient years	35	20.7	35.5	33.4	70.5	54.1
Infections						
*Any infection [n events]*	*109*	*42*	*122*	*77*	*231*	*119*
Mild infection	91	30	79	43	170	73
Moderate infection	17	12	43	32	60	44
Severe infection	1	0	0	2	1	2
Annual rate [number of other infections per patient-year (two-sided 95% CI)]	3.1 (2.0, 4.8)	2.0 (0.9, 4.8)	3.4 (2.2, 5.4)	2.3 (1.0, 4.9)	3.3 (2.4, 4.5)	2.2 (1.2, 3.9)
SBIs [*n* events]	0	0	0	1	0	1
Annual rate [number of SBIs^*^ per patient-year (upper one-sided 99% CI)]	0 (0.13)^†^	0 (0.22)^†^	0 (0.13)^†^	0.03 (0.31)	0 (0.06)^†^	0.02 (0.19)
Absences from work/school, days [rate per person-year]	180 (5.2)	75 (3.6)	72 (2.0)	55 (1.7)	252 (3.6)	130 (2.4)
Hospitalizations due to infection, days [rate per person-year]	29 (0.8)	0	0	10 (0.3)	29 (0.4)	10 (0.2)
Systemic antibiotic use, [*n* (%)]	24 (63.2)	6 (60.0)	25 (67.6)	13 (76.5)	49 (65.3)	19 (70.4)
Annual rate [days on treatment per patient-year (two-sided 95%CI)]	32.1 (17.2, 60.1)	70.6 (22.7, 220.0)	62.6 (31.0, 126.4)	30.9 (12.2, 78.4)	47.2 (28.4, 78.6)	46.0 (21.3, 99.4)

^*^Defined as bacterial pneumonia, bacteremia/septicemia, osteomyelitis/septic arthritis, bacterial meningitis, and visceral abscess.

^†^Calculation for the upper limit of two-sided 95% or one-sided 99% CI was based on the standard Poisson model for zero counts.

Abbreviations: CI, confidence interval; SBI, serious bacterial infection.

**Figure 2:**
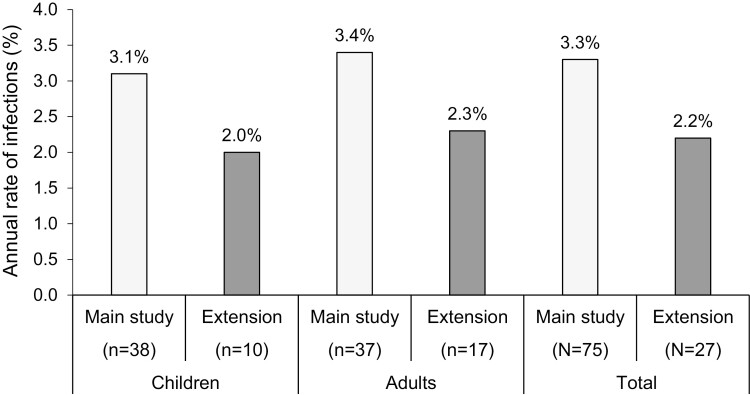
Annual rate of infections in the main study and the extension by age group.

The annual rate of other infections in the main study was 3.3 (95% CI 2.4, 4.5) and in the extension, it was 2.2 (95% CI 1.2, 3.9) infections per patient-year (Table 3). A summary of the annual rates of all infections across the studies and age groups are shown in Fig. 2. The decrease in the rate of infections was observed for both the pediatric and adult populations. Infections were reported by 61 (81.3%) patients in the main study and 25 (92.6%) patients in the extension study (Table 3). Of the total of 231 infections in the main study, five were considered serious adverse events (SAEs) (tracheitis, respiratory syncytial virus, abscess limb, *Pneumocystis jirovecii* infection, and osteomyelitis). Four out of five infections were considered mild or moderate. One acute bronchitis due to respiratory syncytial virus infection was considered severe. Of the 119 infections in the extension, three infections in two patients were considered SAEs: *Escherichia* bacteremia, infected bite, and fever of unknown origin. There were two severe infections: *E. coli* bacteremia (described above) and fever of unknown origin, which was treated with antibiotics despite the absence of strong indicators of bacterial infection and which resolved after 3 days.

In the main study, 49 out of 75 (65.3%) patients received systemic antibiotics throughout the primary observation period, for a rate of 47.2 days of treatment per patient year. A similar proportion of patients received antibiotics in the extension (19; 70.4% of patients). In the main study, a markedly higher rate of days on treatment was observed in the adult population compared with the pediatric population (62.6 vs. 32.1 days). In the extension, the opposite trend was observed: pediatric patients had a rate of 70.6 days on treatment per patient-year vs. the rate for adults of 30.9 days, for an overall rate of 46.0 days on treatment per patient-year (Table 3).

### SCIg dosing and IgG bioavailability

The mean ratio for DCF was 1.278 in the main study, and the population PK model yielded a DCF of 1.33 for a median patient.

The geometric mean of AUC after SCIg administration at PK_SC2_ over the AUC after IVIg administration at PK_IV_ was determined to be 1.0644 (90% CI: 1.0281, 1.1020), thus confirming bioequivalence.

### IgG plasma levels

**Figure 3:**
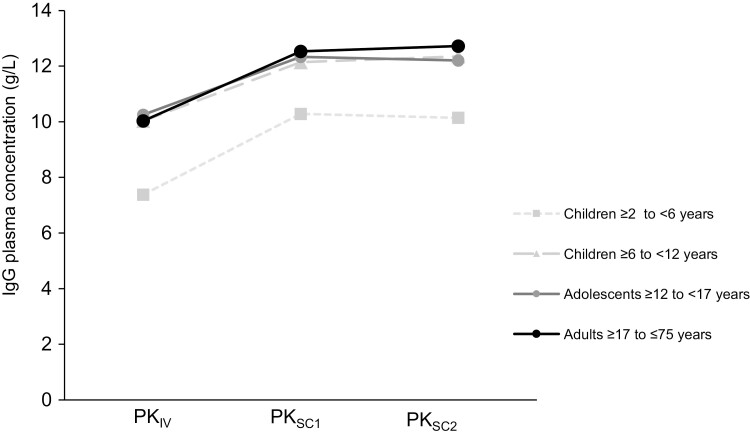
Mean IgG trough concentrations in the main study. *N* = 37. PK_IV_ = PK following IVIg prior to the switch to cutaquig; PK_SC1_ = PK after the 11th infusion of cutaquig; PK_SC2_ = PK after the 28th infusion of cutaquig–at steady state.

IgG plasma levels were evaluated in 37 patients at three timepoints (after IVIg administration before the switch to cutaquig, after the 11th infusion, and after the 28th infusion) within the main study. The mean plasma IgG and IgG subclass trough levels were nearly constant during the course of the study, with higher levels after cutaquig administrations compared with those following IVIg (Fig. 3). There were no patients with IgG trough levels below 5 g/l, and the minimum trough level observed from the second week onwards was 6.1 g/l. There was no indication of marked differences in trough levels by age group, though patient numbers were low in the pediatric subgroup.

### Population PK analysis

**Figure 4:**
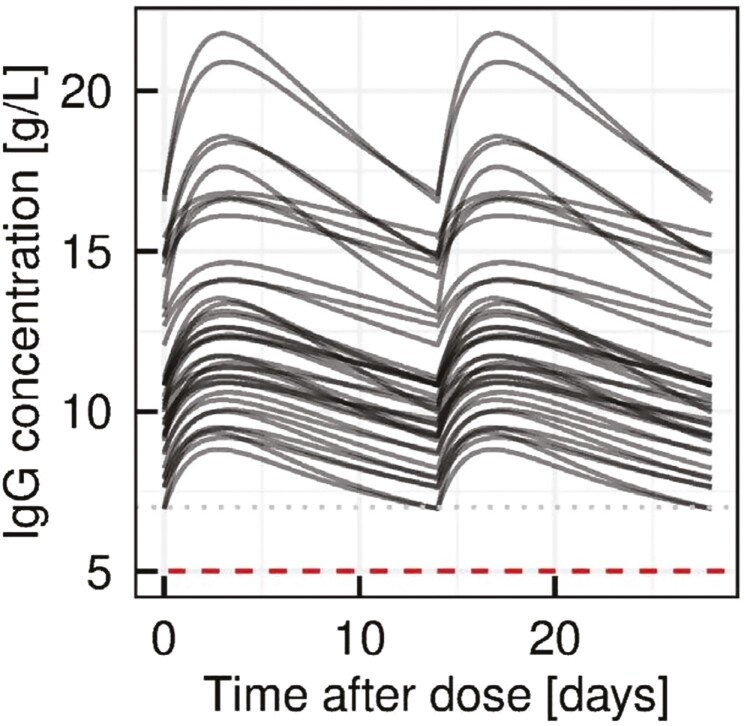
Simulated concentration vs. time plots for a 14-day dosing interval and for a DCF of 1.4 in the main study. IgG concentration was simulated with a 14-day interval for a DCF of 1.4 in all patients enrolled in the PK substudy, demonstrating that IgG levels are not expected to fall below 7.0 g/l. Each black line corresponds to a patient in the PK substudy. The dashed red and grey lines indicate levels of 5.0 g/l and 7.0 g/l, respectively. DCF = dose conversion factor.

In the popPK analysis, simulated IgG concentrations for patients in the PK substudy for the dosing interval of every 2 weeks (using a conversion factor of 1.4) are provided in Fig. 4. Simulated trough concentrations did not fall below 7.0 g/l for any patients with this dosing regimen. Furthermore, for a dosing interval of up to 2 weeks, the median predicted risk for trough concentration values of <5.0 g/l was below 2% (median fraction of the whole population and pediatric patients with a trough concentration of <5 g/l was 1.4 [95%CI 0.2–4.005] and 1.8 [95% CI 0.2–5.605], respectively). The analysis indicated no clinical relevance of the deviations between the populations. Similar results were obtained with a conversion factor of 1.0 (data on file).

The analysis indicated no clinical relevance of the deviations between the populations.

### Treatment compliance

The majority of patients in the main study (60/75; 80.0%) did not have more than two infusions outside the treatment window of ±2 days, and 66.7% of patients administered all infusions within the treatment window. This trend continued into the extension study as most patients (81.5% at study sites and 74.1% during home visits) did not have more than two infusions outside the treatment window. The most common reason for infusion interruptions was technical problems, such as leaks at the pump or syringes. Other reasons included miscalculations of dose and change of infusion day.

### Patient experience

There were no significant changes over time in the mean CHQ-PF50 scores of patients younger than 14 years in either study. The CHQ-PF50 scores in the extension study were modestly favorable, with positive changes for the majority of items over time.

The SF-36 survey was used for patients aged 14 years and older. In the main study, the overall mean scores increased slightly for both summary scores (physical health and mental health) and either increased or remained relatively the same for each of the eight scales. The largest increase was seen in the general health score (mean increase of 3.29 points). In the extension study, there were improvements from the baseline of the extension to the end-of-study visit in the mean scores for summary physical health for five of the eight scales (physical functioning, bodily pain, vitality, social functioning, and mental health). The largest improvement from baseline was observed in the bodily pain score (3.14 points). There were also small improvements in the physical and mental health summary scores, with increases of 1.09 and 0.30 points, respectively.

### Safety and tolerability

In the main study, 11 (14.7%) patients experienced 14 systemic AEs (excluding ISRs and infections) that were related to cutaquig. In the extension, two (7.4%) patients experienced seven related AEs (Table 4). There were seven SAEs in six (8.0%) patients in the main study and 13 SAEs in seven (25.9%) patients in the extension; none of these SAEs was considered related to the study medication. There were no AEs leading to death or withdrawal or other significant AEs in either study.

**Table 4:** Adverse events related to cutaquig

MedDRA Preferred Term	Children (≥2 to <17 years)		Adults (≥17 years)		All patients
	Main study (*N* = 26)	Extension (*N* = 8)	Main study (*N* = 37)	Extension (*N* = 17)	Both studies (*N* = 102)
	*N* (%) *n*	*N* (%) *n*	*N* (%) *n*	*N* (%) *n*	*N* (%) *n*
*Any related AE*	*4 (15.3) 5*	*0*	*7 (18.9) 9*	*2 (11.8) 7*	*13 (12.7) 21*
Headache	1 (3.8) 1	–	1 (2.7) 2	2 (11.8) 3	4 (3.9) 6
Pyrexia	–	–	1 (2.7) 1	1 (5.9) 1	2 (2.0) 2
Chills	–	–	*–*	1 (5.9) 2	1 (1.0) 2
Abdominal distension	–	–	1 (2.7) 1	–	1 (1.0) 1
Abdominal pain upper	–	–	1 (2.7) 1	–	1 (1.0) 1
Body temperature increased	1 (3.8) 1	–	–	–	1 (1.0) 1
Coombs’ direct test positive	–	–	1 (2.7) 1	–	1 (1.0) 1
Free hemoglobin present	–	–	1 (2.7) 1	*–*	1 (1.0) 1
Haptoglobin decreased	–	–	1 (2.7) 1	–	1 (1.0) 1
Hemoglobin increased	–	–	1 (2.7) 1	–	1 (1.0) 1
High free hemoglobin	1 (3.8) 1	–	–	*–*	1 (1.0) 1
Myalgia	1 (3.8) 1	–	–	–	1 (1.0) 1
Nausea	–	–	–	1 (5.9) 1	1 (1.0) 1
Vomiting	1 (3.8) 1	–	–	–	1 (1.0) 1

Abbreviations: AE, adverse event; *N*, number of patients with AEs; MedDRA, Medical Dictionary for Regulatory Activities, n, number of events.

When analyzed by age, the most common systemic adverse reactions (defined as adverse events occurring during or within 72 h of infusion or any adverse events otherwise causally related) were asthma, cough, and vomiting, each experienced by 10.5% of children. Headache was by far the most common systemic AE recorded for the adult cohort (18.6% of patients), followed by fever, dermatitis, and diarrhea, each experienced by 11.6% of adult patients (Table 5). When systemic AEs were analyzed as a function of infusions, infusions were most commonly associated with asthma, cough, and vomiting in children and headache and diarrhea in adults (rate of 0.002 each).

**Table 5:** Adverse reactions^*‡^ in ≥5% of pediatric and adult patients and rate per infusion (both studies)

Preferred Term	Children (≥2 to <17 years)		Adults (≥ 17 years)	
	Number (%) of patients (*N* = 38)	Number (rate†) of ARs (*N* = 3283 infusions)	Number (%) of patients (*N* = 43)	Number (rate†) of ARs (*N* = 3956 infusions)
Local reaction	28 (73.7)	442 (0.14)	31 (72.1)	648 (0.16)
Systemic ARs				
Asthma	4 (10.5)	7 (0.002)	–	–
Cough	4 (10.5)	5 (0.002)	–	–
Vomiting	4 (10.5)	5 (0.002)	–	–
Nasal congestion	3 (7.9)	4 (0.001)	–	–
Fever	3 (7.9)	4 (0.001)	5 (11.6)	5 (0.001)
Headache	3 (7.9)	3 (0.001)	8 (18.6)	10 (0.002)
ALT increased	3 (7.9)	3 (0.001)	–	–
Leukopenia	3 (7.9)	3 (0.001)	–	–
Neutropenia	3 (7.9)	3 (0.001)	–	–
Dermatitis	2 (5.3)	3 (0.001)	5 (11.6)	6 (0.001)
Oropharyngeal pain	2 (5.3)	3 (0.001)	–	–
Urticaria	2 (5.3)	2 (0.001)	–	–
AST increased	2 (5.3)	2 (0.001)	–	–
Abdominal pain	2 (5.3)	2 (0.001)	–	–
Ear pain	2 (5.3)	2 (0.001)	–	–
Diarrhea	–	–	5 (11.6)	7 (0.002)
Muscle spasms	–	–	4 (9.3)	5 (0.001)
Back pain	–	–	4 (9.3)	4 (0.001)

*Excluding infections.

‡Adverse reactions were defined as adverse events occurring during or within 72 h of infusion or any adverse events otherwise causally related.

†Rate = total number of adverse reactions divided by total number of infusions.

ALT, alanine aminotransferase; AR, adverse reaction; AST, aspartate aminotransferase; *N*, number of patients who experienced an AR that was experienced by ≥5% of the respective population.

The laboratory results did not indicate any safety concerns in either study. Five patients had positive Coombs’ tests after screening. None of the patients who had a confirmed positive Coombs’ test also had a drop in hemoglobin of ≥2 g/dl, and therefore there was no indication of hemolysis during the studies. There were no cases of documented viral transmission.

The majority of patients presented with blood pressure, pulse rate, respiratory rate, and temperature within the normal range, with little fluctuation in mean values throughout the main study and the extension. Therefore, the administration of cutaquig did not appear to influence vital sign values.

### Infusion site reactions

**Figure 5:**
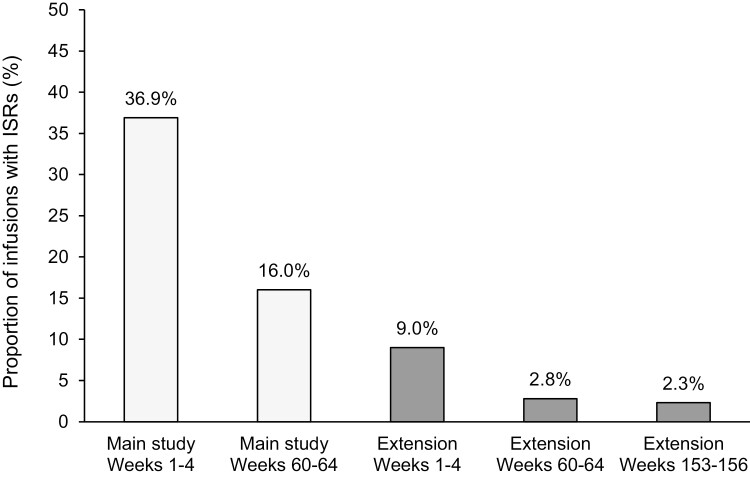
Proportion of infusions with ISRs over time. ISR, infusion site reaction.

Of the 7239 cutaquig infusions administered over both studies, 15.0% (1,085 infusions) were associated with an ISR. The rates of ISRs declined over time. The proportion of infusions with ISRs was highest during the first 4 weeks in the main study (36.9%). Over the duration of the study, the incidence of ISRs decreased to 16% in the last 4 weeks of the study. This trend continued into the extension, with the proportion of infusions with ISRs declining from 9% during the first 4 weeks of the extension to 2.3% in the last 4 weeks (Fig. 5). The most drastic decrease from the main study to extension study was seen in the number of infusions associated with mild ISRs (20.1% of infusions in the main study to 2.0% of infusions in the extension).

**Figure 6:**
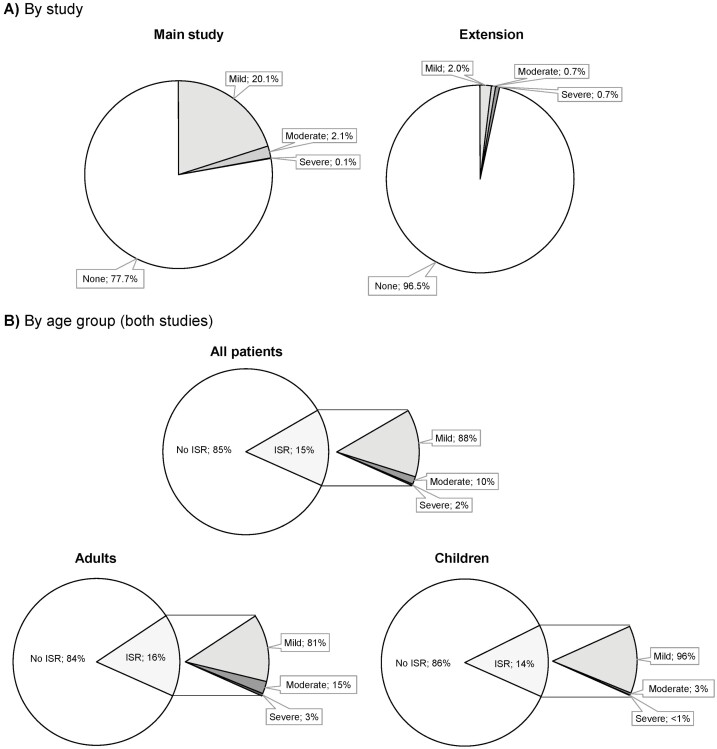
Proportions of infusions with ISRs by severity. Mild = AE, usually transient, which causes discomfort but does not interfere with the patient’s routine activities; moderate = an AE which is sufficiently discomforting to interfere with the patient’s routine activities; severe = an AE which is incapacitating and prevents the pursuit of the patient’s routine activities.

Over the whole main study period, 77.7% of the 4462 infusions resulted in no ISRs. The majority of the ISRs were mild (20.1% of all infusions). In the extension study, most of the 2777 infusions (96.5%) induced no ISRs (Fig. 6A).

When combined for both studies, 15% of infusions were associated with reactions. Of this 15%, most of the ISRs were mild (88% of ISRs; 952 infusions) and moderate (10% of ISRs; 112 infusions). Only 2% of ISRs (23 infusions) were associated with severe reactions (Fig. 6B). One patient experienced severe ISRs over 18 infusions in the extension study. The reactions included hives, swelling, and “other” (including wheals) and all were located on the abdomen. Despite this, the patient still completed the study with 96 infusions. When analyzed by age, the rate of local reactions in children and adults was 0.14 and 0.16, respectively (Fig. 6B and Table 5). In both age groups mild ISRs were by far the most common, and in children mild ISRs made up 96% of local reactions.

Overall, the mean proportion of patients who experienced at least one ISR declined from 73.3% in the main study to 44.4% in the extension study. The greatest decrease was observed in the proportion of patients experiencing mild reactions (from 72.0% to 37.0%). The same trend was seen also for the proportion of patients who experienced moderate reactions which decreased from 25.3% to 18.5%. Severe reactions were observed in four patients in the main study and three patients in the extension, and only one patient (discussed above) experienced severe ISRs with more than a single infusion in the extension study.

## Discussion

We present here the results from the pivotal studies with 16.5% SCIg cutaquig that evaluated the long-term treatment of patients with PIDs, with more than four years of follow-up for nearly half of the patients. The duration of the main study was up to 70 weeks, with a mean follow-up of 60 weeks (due to some patients withdrawing early). The duration of the extension study was 168 weeks, with a mean follow-up time of 104 weeks. In total, 7239 infusions of cutaquig were administered over up to 238 weeks in the two studies. To our knowledge, this is one of the longest prospective follow-ups of PID patients treated with a SCIg.

The long-term follow-up allowed us to evaluate the tolerability and efficacy that can be expected from cutaquig treatment with systematic monitoring and good compliance. It is notable that in the extension a high proportion of patients received all of their infusions within the treatment window, regardless of whether they were administered at the clinic or at home (81.5% and 74.1% of patients, respectively).

SCIgs are generally well tolerated, and in line with this, our study showed an overall low rate of ISRs. Although local reactions at the site of injection were recorded for 36.9% of infusions in the first four weeks, their incidence decreased over the duration of the study to 16% in the last four weeks. Importantly, over the duration of the extension, the incidence of ISRs decreased from 9% in the first four weeks to 2.3% in the last four weeks. While this lower rate at the start of the extension may be attributed to patients with more tolerable ISR profiles in the main study continuing into the extension, the trend of decreasing frequency of ISRs over time suggests good tolerability of cutaquig with up to 238 weeks of treatment.

In addition, the vast majority of the ISRs were mild. Most of the reactions were consistent with those observed for any injectable therapy (e.g. redness, swelling, and pruritus). The rate of severe ISRs remained below 1% in both studies. In the extension, one participant experienced 18 severe reactions (of the 23 severe ISRs in the study). This participant nevertheless went on to receive 96 infusions in the study; furthermore, no participant discontinued the study due to tolerability issues. Therefore, our results support the notion that local reactions after cutaquig infusions are rarely severe and that their incidence and severity decrease with subsequent infusions. The decrease in the rate of ISRs may be attributed to decreased tissue responsiveness over time or improved injection technique, supporting the notion that local reactions to cutaquig were largely well tolerated once the patients or their caregivers got accustomed to infusing the product.

The decrease in ISRs was not caused by the adjustment of dosings, such as lowering the speed of infusion or the dose. In fact, while the dose remained unchanged, the mean maximum infusion flow rate increased from 60.4 ml/h to 97.9 ml/h and the mean infusion time consequently decreased from 113.3 min to 76.4 min in the adult population over time, without an increase in local reactions. Compared with Hizentra®, which is administered at the maximum volume of 25 ml/site and infusion rate of up to 25 ml/h/site [16], cutaquig allows for a maximum volume in adults and children of 40 ml/site and up to 29 ml/site, and a maximum rate of 52 ml/h/site and 25 ml/h/site, respectively. The benefit of a higher infusion rate to the patients is shorter infusion times and therefore decreased treatment burden and more autonomy.

The incidence of related systemic adverse reactions was also low. Importantly, the incidence of systemic reactions did not increase with prolonged treatment and, in fact, was lower in the extension than in the main study (7.4% vs. 14.7%, respectively). The most common systemic adverse reaction was a headache, and none of the adverse events related to cutaquig were serious. There was no evidence of thromboembolisms, hemolysis, or hemolytic anemia, even with long-term treatment. Therefore, the high rate of infusion coupled with the overall low rate of ISRs and low incidence of related systemic adverse reactions suggest favorable safety and tolerability of cutaquig with long-term treatment.

The main study met its primary endpoint, with no SBIs. During the extension study, one elderly patient experienced *E. coli* bacteremia, resulting in the rate of SBIs per person-year of 0.02 (upper 99% CI 0.91) in the extension. This is well below a rate of 1.0 SBI per person-year, which, according to the FDA is adequate to provide substantial evidence of efficacy [17].

A favorable rate of non-serious infections (3.432 infections per person-year) for patients treated with cutaquig was first reported in the interim analysis of the main study data [13]. The final analysis of the data supports this observation, with a rate of 3.3 infections per person-year. In the extension, the rate of non-serious infections was 2.2 per person-year, showing that the efficacy of cutaquig is maintained with prolonged treatment. Influencing factors for the decrease in the infection rate might be the high treatment compliance as well as the long duration of follow-up, which allows examination of infection rates over seasonal fluctuations rather than as a snapshot of a few months. It cannot be ruled out that those patients who already had good infection prevention in the main study chose to continue into the extension and thus biased the outcome observed in the extension study. However, it may also be argued that careful, regular monitoring and good compliance resulted in continued control of infections and complications.

The infection rate observed with cutaquig is in line with previously published rates for other SCIg products [11, 18, 19]. Notably, the infection rate with cutaquig was two times lower than the 5.18 infections per person-year reported for the 20% SCIg (Hizentra) in a prospective study of 51 patients observed for 40 weeks [20]. However, this is the first report to prospectively show that efficacy rates are maintained for up to four years of follow-up.

The efficacy of cutaquig was also supported by the low number of days in the hospital and away from work/school in the main study, which further declined in the extension, likely reflective of the decrease in the incidence of infections. These outcomes suggest an important reduction in the disruption to patient lives and are complemented by favorable patient experience outcomes in the form of modestly improved mean scores for 5 of the 8 scales in the extension study. Previously published data for long-term SCIg use in patients with PID with the 20% SCIg Hizentra and 16% SCIg^®^ also showed relatively stable SF-36 scores over 72 weeks and 24 months of treatment, respectively [21, 22].

The PK substudy showed that IgG plasma trough levels were nearly constant during the course of the evaluation. PK data from the patients in the main study was used in a popPK modelling analysis of different SCIg dosing regimens. Cutaquig dosing is based on the patient’s previous IVIg dose, with a conversion factor of 1.30. The conversion factor was calculated based on the mean ratio of the PK substudy (DCF 1.278). The popPK modelling confirmed this to be an appropriate conversion factor, with the calculated DCF of 1.33 (for a median patient). Furthermore, the popPK simulation for every-other-week dosing of cutaquig and a DCF of 1.4 (as well as a DCF of 1.0) showed that IgG trough levels can be expected to remain well above the lower limit of 5 g/l, which is considered a minimum target for infection prevention in PID. This is in line with popPK analyses with other SCIg products [23, 24]. Therefore, this simulation supports the every-other-week cutaquig treatment with the approved DCF of 1.30. Confirmation of the feasibility of every-other-week cutaquig dosing in patients with PIDs is currently ongoing in a clinical study.

In the long-term, there is no evidence to suggest that adjustment of cutaquig dose is needed once the dose has been calculated using the approved DCF. In the extension study, patients remained on the same dose of SCIg they had been receiving in the main study with no loss of effectiveness over time. De novo enrolled patients continued receiving the dose of their previous SCIg, with no apparent detriment with respect to protection from infection.

A limitation of all clinical IVIg and SCIg studies is the use of concomitant systemic antibiotics to help control any breakthrough infections. It should be noted, however, that periodic antibiotic treatment alone is not enough to control infections and prevent complications, as evidenced by high morbidity and mortality rates in PID patients before the widespread use of IgG replacement therapy. In the clinic, as in the clinical studies, many PID patients, despite receiving IgG replacement, will require antibiotic treatment at some point in their treatment journey. In line with this, 65 to 70% of patients in our studies received systemic antibiotics and antibiotic use remained relatively stable over time. Analysis by age groups revealed that while the duration of antibiotic use increased in the pediatric population, it decreased in the adult population. However, it is difficult to draw any conclusions from these trends, and it is likely that these differences are indicative of the individual patient’s clinical situation and provider treatment preferences. Another limitation of the study is the inclusion of the six de novo patients in the extension study. However, the data from these patients did not markedly affect the overall length of follow-up and no SBIs or new safety issues were noted in these patients.

In conclusion, in this first examination of long-term use of cutaquig, we show that its prolonged use of up to 238 weeks in the home setting is feasible and well tolerated by both pediatric and adult patients with PIDs. Cutaquig was shown to convey effective protection from a serious infection, and low rates of local and systemic adverse reactions support the favorable safety profile of cutaquig.

## Data Availability

The data that support the findings of these studies are available on request from the corresponding author. The data are not publicly available due to their containing information that could compromise the privacy of research participants.

## Abbreviations

AE: adverse event
AUC: area under the curve
AUCτ: AUC standardized to 1 week
CHQ-PF50: Child Health Questionnaire-Parent Form, 50 items
CVID: common variable immunodeficiency
DCF: dose conversion factor
EMA: European Medicines Agency
ESID: European Society for Immunodeficiencies
FDA: the US Food and Drug Administration
IDMC: independent Data Monitoring Committee
Ig: immunoglobulin
IRB: institutional review board
ISR: infusion site reaction
IV: intravenous(ly)
PAGID: Pan-American Group for Immunodeficiency
PID: primary immunodeficiency
PK: pharmacokinetic(s)
PopPK: population PK
QoL: quality of life
SAE: serious adverse event
SBI: serious bacterial infection
SC: subcutaneous(ly)
SD: standard deviation
SF-36: Short Form Health Survey, 36 item
TEAE: treatment-emergent adverse event
XLA: X-linked agammaglobulinemia

## Clinical Trial Registration

Trial registry identification NCT01888484 (SCGAM-01) and NCT03907241 (SCGAM-03) (www.clinicaltrials.gov).
